# Age-related brain atrophy in cats without apparent neurological and behavioral signs using voxel-based morphometry

**DOI:** 10.3389/fvets.2022.1071002

**Published:** 2022-11-24

**Authors:** Yuji Hamamoto, Yoshihiko Yu, Rikako Asada, Satoshi Mizuno, Daisuke Hasegawa

**Affiliations:** ^1^Veterinary Medical Teaching Hospital, Nippon Veterinary and Life Science University, Musashino, Japan; ^2^Laboratory of Veterinary Radiology, Faculty of Veterinary Science, Nippon Veterinary and Life Science University, Musashino, Japan; ^3^The Research Center for Animal Life Science, Nippon Veterinary and Life Science University, Musashino, Japan

**Keywords:** aging, feline, MRI, parietal cortex, VBM, brain atrophy

## Abstract

**Introduction:**

Brain atrophy is observed with aging and may cause cognitive decline or dysfunction. Aging cats may demonstrate behavioral changes related to cognitive dysfunction. In the clinical veterinary field, although the conventional region of interest method by manual or semiauto tracing on magnetic resonance imaging is used to detect atrophy of regional structures, such as the hippocampus, it is difficult to assess atrophy globally. Voxel-based morphometry (VBM) has been developed to detect global and regional abnormalities in humans. The purpose of the present study investigates whether the feline brain volume decreases with aging using VBM analysis.

**Materials:**

A total of 65 cats, aged 17–200 months, without apparent neurological and behavioral signs were included in the statistical analysis.

**Results:**

We observed that the gray matter in the bilateral parietal lobes was decreased significantly with aging. The regions that showed decreased volume included the right postcruciate, cingulate gyrus, rostral suprasylvian/ectosylvian gyri, and the left postcruciate gyrus. No significant reduction in white matter was observed. Together, our results show that age-related brain atrophy can be detected using VBM analysis.

**Discussion:**

The age-related atrophy of the parietal cortex may not cause neurological and behavioral signs in cats. Therefore, veterinarians should consider age when assessing the relation between morphometric and functional abnormalities of the parietal cortex in cats.

## Introduction

Aging cats sometimes show various neurological and behavioral changes (1). Since behavior is dependent upon brain function, age-related behavioral changes include many neurological abnormalities. Although brain atrophy with aging is considered one cause of cognitive decline or dysfunction, age-related behavioral changes in cats often appear with signs of other illnesses such as hypertension, hyperthyroidism, pain, and separation anxiety (1). When aged cats with behavioral changes have no other medical problems, the cats are diagnosed with cognitive dysfunction (2). Therefore, veterinarians may have to perform many clinical examinations to identify the cause of age-related behavioral changes. However, it is difficult to accurately assess physical and/or neurological examinations in cats, since many cats are very stressed during clinic visits (3). Additionally, a diagnosis of cognitive decline or dysfunction should rule out other diseases, since it is extremely difficult to obtain brain tissue for pathological diagnosis antemortem. For these reasons, accurate identification of the cause of neurological and behavioral changes with aging may be difficult.

Voxel-based morphometry (VBM) was developed as a statistical morphometric brain analysis tool in humans using magnetic resonance imaging (MRI) (4). VBM analysis can evaluate reproducibly the decreased brain area through volume and signal intensity on MRI. In human medicine, this technique has been used to characterize subtle changes in Alzheimer's disease (5), Parkinson's disease (6), epilepsy (7), and psychological disorders (8). Aging studies using VBM analysis have been reported in some animal models such as mice (9), rats (10), dogs (11), rhesus macaques (12), and chimpanzees (13). In contrast, although cats are often used in neuroscientific studies and have a relatively long lifespan, no report has used VBM analysis on the feline brain to assess age-related changes.

Pathological changes similar to that in human brain aging have been observed in the hippocampus of aged cats (14). These pathological changes are known as neurofibrillary tangles, which form as thick bundles near the cell surface of affected neurons (15). Additionally, the neurofibrillary tangles are also one of the pathological changes caused by Alzheimer's disease in humans. Thus, it has been considered that cats may be effective model animals for Alzheimer's disease since neurofibrillary tangles are not observed in dogs (16) and monkeys (17). Clinically, hippocampal atrophy on MRI can be used as a biomarker for Alzheimer's disease in humans (18). Hippocampal atrophy is observed in cats with spontaneous temporal lobe epilepsy (19, 20) and hippocampal sclerosis, as observed in pathology, is present in cats with structural epilepsy (21, 22). In contrast to canine studies (23–26), however, no report has compared the hippocampal volume in old cats with that in young cats. Therefore, an investigation into feline hippocampal changes with aging on MRI is needed to diagnose abnormal hippocampal structure accurately.

We hypothesized that aging may induce physiologic atrophy in whole brain and/or brain regions even though cats may not show any neurological and behavioral signs. The purpose of the present study is to assess morphometric brain changes with aging in cats without neurological and behavioral signs. Therefore, we retrospectively collected brain MRIs of cats without neurological and behavioral signs at various ages from medical records and investigated brain atrophy with aging using VBM analysis. The results from the present study are helpful to consider physiologic brain atrophy with aging when diagnosing diseases with subtle changes, such as cognitive decline or dysfunction, in the whole brain and brain regions.

## Materials and methods

### Study design and subjects

**Figure 1:**
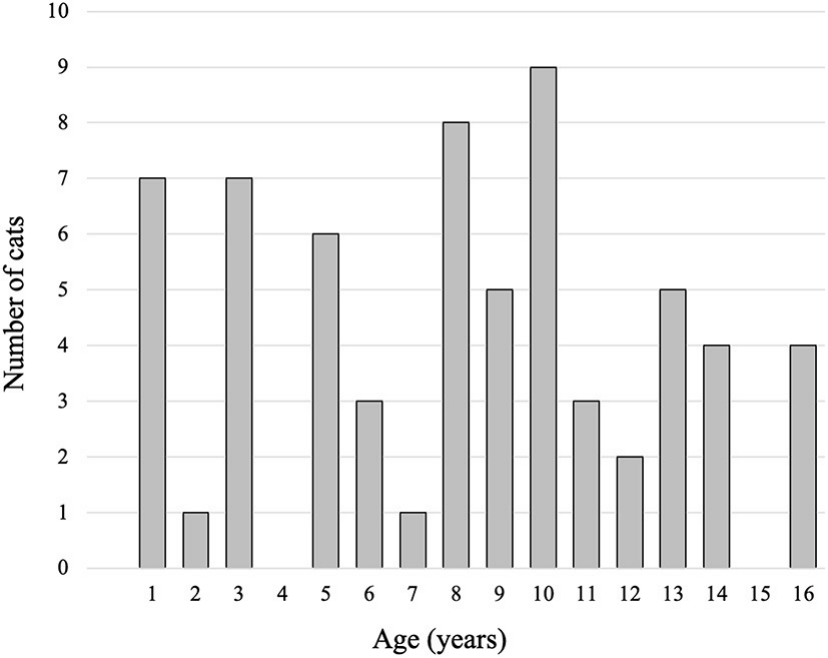
Histogram showing the age distribution of cats in the present study.

This is a retrospective analysis using clinical and laboratory data. Brain MRIs from cats with pathogenic intracranial structural lesions, intracranial neurological signs including epileptic seizures, and/or behavioral abnormalities were excluded. MRI data sets from 65 cats (36 males, 12 intact; 29 females, 14 intact) were included in the present study. Age ranged from 16 to 200 months (median age, 106 months), and body weight was between 2.1 and 5.9 kg (median weight, 4.4 kg). The age distribution of the cats is shown in Figure 1. Cat brain MRI data were obtained from 39 clinical scans and 26 laboratory scans. The clinical data set was obtained as routine diagnostic imaging work between April 2015 and March 2021 from the Veterinary Medical Teaching Hospital of Nippon Veterinary and Life Science University (Tokyo, Japan). The breed distribution included 30 domestic shorthair cats, two Norwegian forest cats, one each of Abyssinian, American shorthair, Bengal, Maine Coon, Russian Blue, Scottish Fold, and Tonkinese. These cats were diagnosed with nasal tumor (*n* = 14), nasopharyngitis (*n* = 8), otitis media (*n* = 4), nasopharyngeal constriction (*n* = 4), external auditory canal tumors (*n* = 2), mandibular gland tumor (*n* = 1), maxillary tumor (*n* = 1), ischemic myelopathy (*n* = 1), immune-mediated polyarthritis (*n* = 1), and no lesions (*n* = 3). All owners of the cats included in this study agreed to the use of their cats' data for academic education and studies and signed a consent form at the first presentation to the teaching hospital. The laboratory data set was obtained between April 2013 and March 2021 from laboratory domestic shorthair cats at the Laboratory of Veterinary Radiology, Nippon Veterinary and Life Science University (Tokyo, Japan) in order to use as control images for epilepsy studies. The MRI study using laboratory cats was approved by the Animal Care and Use Committee of Nippon Veterinary and Life Science University.

### Imaging pre-procedures

All MRIs were obtained using a 3.0-Tesla system (Signa HDxt 3.0T, GE Healthcare, Chicago, IL, USA) with an 8-ch human knee array radio frequency coil. The cats were anesthetized with propofol for tracheal intubation, maintained with inhalation of an isoflurane and oxygen gas mixture. During imaging procedures, the cats were in prone position with their heads placed in the radio frequency coil. Although various sequences were obtained depending on the individual, after confirming that no structural lesions were observed in the brain, only 3-dimensional T1-weighted images using a spoiled gradient recalled acquisition in the steady state sequence (3D T1W SPGR; repetition time = 6.5 ms, echo time = 3.1 ms, field of view = 15 × 15 cm, slice thickness = 0.6 mm, slice gap = 0 mm, matrix = 256 × 192, number of excitation = 1, and scan time = 325 s) was used for the VBM analysis.

### Software for VBM analysis

VBM analysis was performed using Matlab 6.5 (Mathworks, Natick, MA, USA) and Statistical Parametric Mapping (SPM12; Wellcome Department of Cognitive Neurology, London, http://www.fil.ion.ucl.ac.uk/spm/). The pre-processing of the VBM analysis was also performed using MRIcroGL (http://www.mccauslandcenter.sc.edu/mricrogl/home) and dcm2nii (https://people.cas.sc.edu/rorden/mricron/dcm2nii.html).

### Image processing

All digital imaging and communications in medicine (DICOM) files were converted to the neuroimaging informatics technology initiative (NIfTI) format using dcm2nii prior to pre-processing. Cats with errors in image processing were excluded from statistical analysis.

All images were aligned in stereotaxic space along the anterior commissure and posterior commissure line using SPM12. The aligned images were rescaled to an isotropic voxel size of 0.3 mm edge length, followed by stripping extra tissue manually using MRIcroGL. The stripped images were segmented into gray matter (GM), white matter (WM), and cerebrospinal fluid (CSF) segments by the feline tissue probability maps (27) using SMP12. In addition, diffeomorphic anatomical registration using exponentiated lie algebra (DARTEL) processing was performed using these initial segmented images. These images were used as the first tissue probability maps.

To create GM and WM data for statistical analysis, all isotropic aligned scans were segmented into GM, WM, and CSF by the first tissue probability maps using SMP12. Furthermore, DARTEL processing was again performed for the GM and WM using the data from the secondary segmentation. GM and WM were warped by 6th-degree B-spline interpolation using the flow field from the secondary DARTEL processing and modulated to be rescaled by the Jacobian determinants of the deformations. Finally, the modulated images were smoothed with 3 mm full width at half maximum isotropic Gaussian kernel for the analysis, and global GW and WM volume were calculated, respectively. Total brain volume was calculated by adding the global GW and WM volume.

### Statistical analysis

#### The global analysis

To investigate the correlativity of aging with the global volume in GM and WM, Spearman's rank correlation coefficient using Easy R (EZR) (28) was performed for the cats' age and the GM and WM volume of each cat was calculated by the segmentation procedure. Statistical significance was defined as *p* < 0.05.

#### The regional analysis

A multiple regression analysis using SPM12 was used to assess brain region reduction with aging in GM and WM, respectively. In the analysis, the total brain volume was used to correct each brain size. The initial voxel threshold for the uncorrected *p*-value of the peak level was set to 0.001. Results were considered a significant reduction when falling below a family-wise error corrected *p*-value of 0.05. In addition, a linear regression model using EZR was performed to examine the significant region. Significant regions were identified according to the atlas of the feline brain (29). In the present study, CSF volume analysis was not conducted since the segmentation of feline sulcal and ventricular CSF volume may be inaccurate. Additionally, analysis was performed without consideration of sex because there was a different proportion of neutered cats in each sex and there was no information on when sterilization was performed in some cats.

## Results

**Figure 2:**
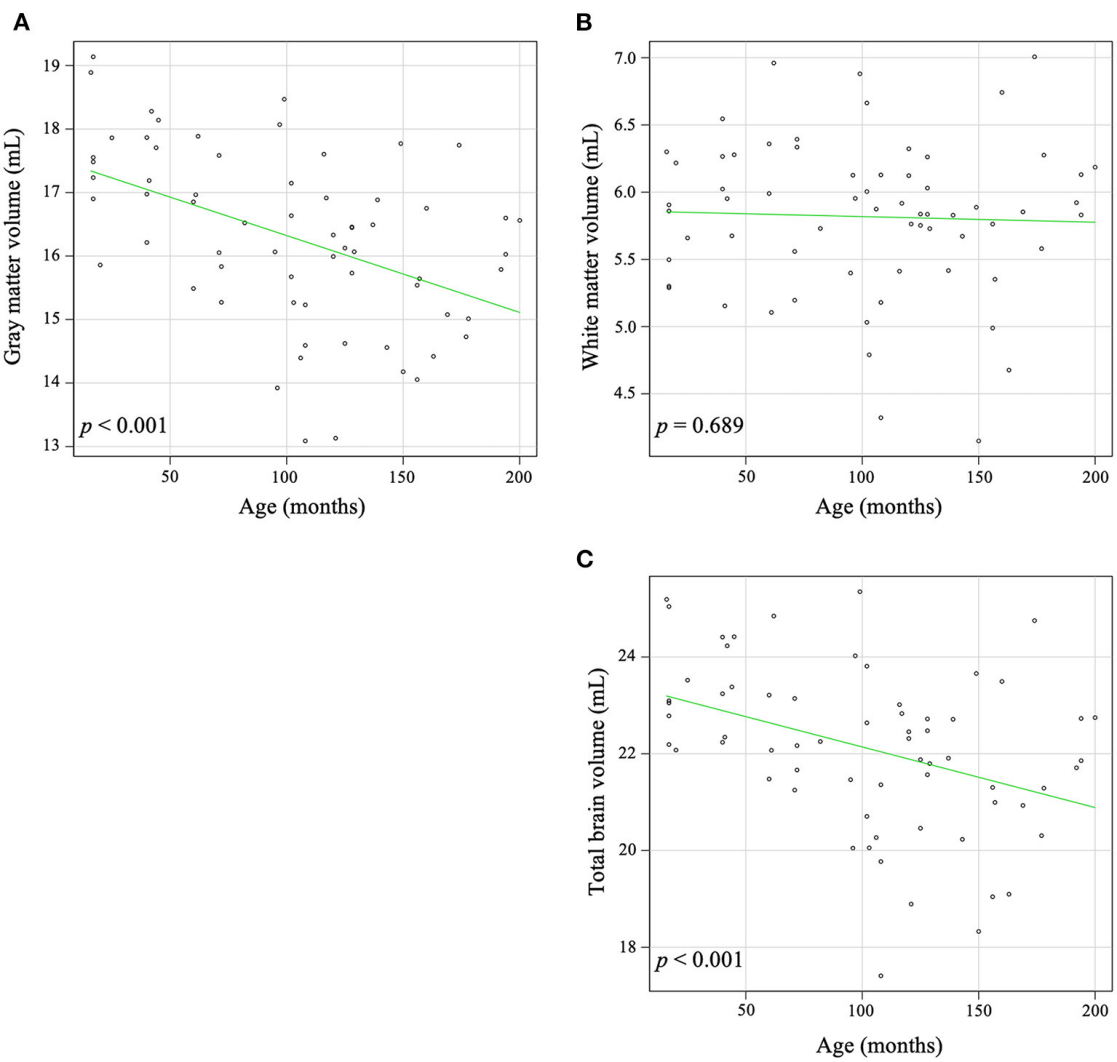
Correlation between age and volume in gray matter **(A)**, white matter **(B)**, and total brain **(C)**. Increasing age was significantly associated with a decrease in gray matter (*r* = −0.491, *p* < 0.001) and the total brain volume (*r* = −0.416, *p* < 0.001). There is no significant correlation between aging and white matter volume (*r* = −0.050, *p* = 0.689).

In the global analysis, increasing age was significantly correlated with a decrease in global GW volume (*r* = −0.491, *p* < 0.001) and total brain volume (*r* = −0.416, *p* < 0.001), but there was no significant correlation between increasing age and global WM volume (*r* = −0.050, *p* = 0.689) (Figure 2).

**Figure 3:**
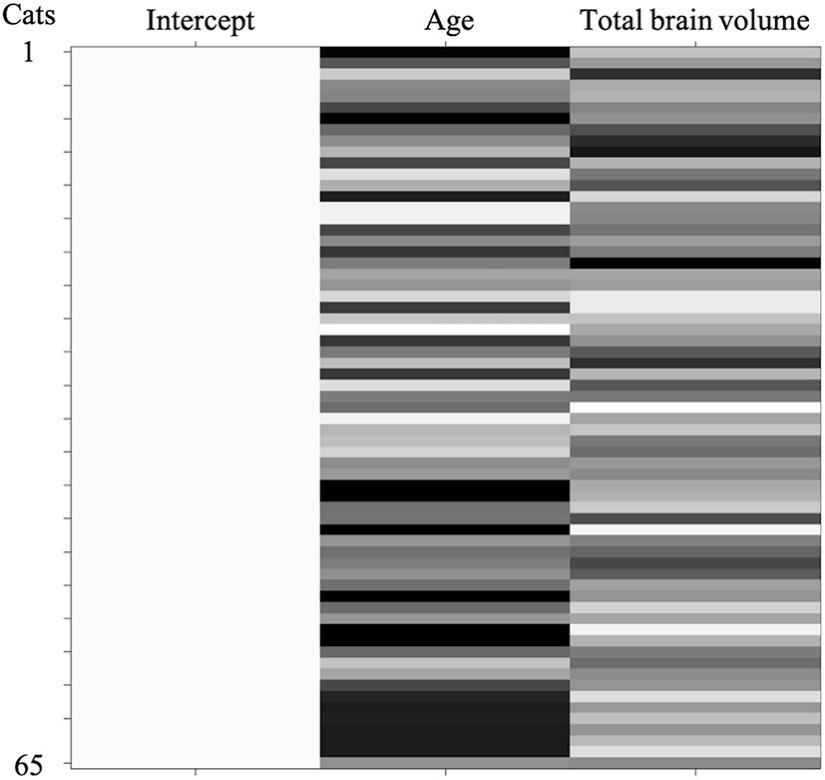
Design matrix of the regional analysis corrected by total brain volume in SPM12 to evaluate aging changes in gray and white matter atrophy. A total number of cats in each condition is displayed along the ordinate and variables are represented in the abscissa. Three columns model the effects of intercept, age, and total brain volume. Age and total brain volume were used as a covariate. Values of each variable **(columns)** for each cat **(rows)** are represented by a grayscale color scheme.

**Figure 4:**
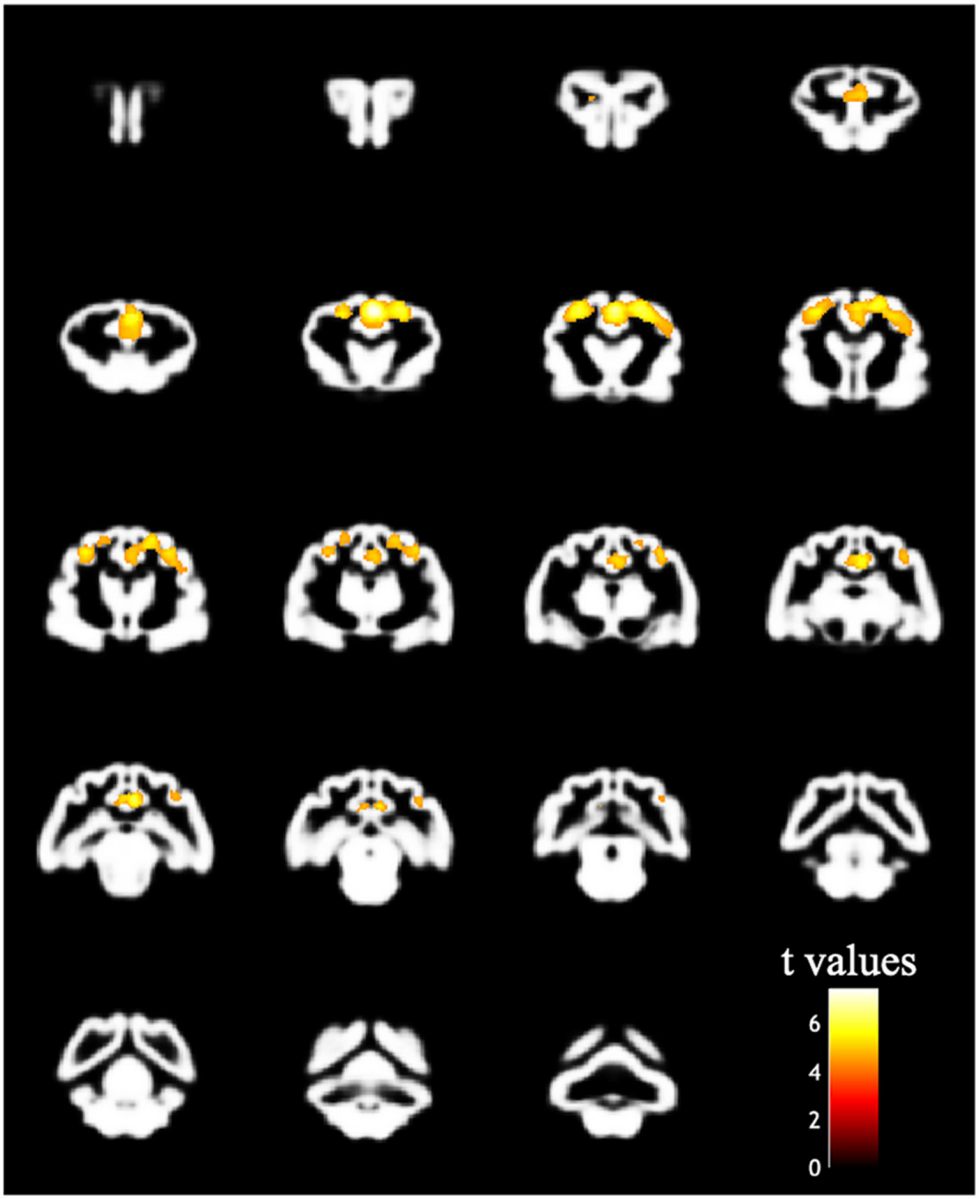
Result of the VBM analysis in the regional analysis. The colored regions indicate that the family-wise error corrected *p* value is < 0.05. The decreased bilateral parietal cortex correlated with increased age.

The design matrix of regional VBM analysis for feline aging in GM and WM is shown in Figure 3. Regional clusters indicating decreased volume were observed in the right and left parietal lobes. The right parietal lobe cluster included the right postcruciate gyrus, the right rostral marginal gyrus, and the right suprasylvian gyrus and spread to the left and right cingulate gyrus and the right ectosylvian gyrus. The peak level point of the right parietal lobe cluster was observed in the right postcruciate gyrus. The left parietal lobe cluster included the postcruciate gyrus and rostral marginal gyrus, and the peak level point of the left parietal lobe cluster was observed in the left postcruciate gyrus. Details of these results are shown in Table 1 and Figure 4. There was no significant regional decrease in WM.

**Table 1:** Details of the clusters that showed a significant decrease with aging in gray matter.

***k***	***t*** **value**	***p*** **(FWE corrected)**	**Coordinates (mm)**	**Side**	**Structure**	**Major brain region**
6,177	7.46	< 0.001	0.6, 3.8, 13.7	Right	Postcruciate gyrus	Parietal
	6.24	< 0.001	1.5,−9.5, 11.0	Right	Cingulate gyrus	Parietal
	5.41	0.002	9.4,−2.4, 12.5	Right	Rostral suprasylvian gyrus / Ectosylvian gyrus	Parietal / Temporal
1,382	6.05	< 0.001	−7.8, 1.6, 13.5	Left	Postcruciate gyrus	Parietal

Cluster size represents the number of significant voxels within each cluster (k coefficient). Coordinates indicate distance from the anterior commissure. FWE, family-wise error.

## Discussion

In the present study, we demonstrated a correlation between feline brain atrophy and aging using VBM. A feline standard brain template was created previously using 7-Tesla MR images (27), and the first feline VBM study was performed in cats with suspected genetic epilepsy (20). However, these previous studies did not investigate changes with aging. In addition, although DARTEL was developed as an algorithm to normalize subject brain images to a brain template accurately in human VBM analyses, previous veterinary studies did not use the DARTEL algorithm (30). Therefore, the present study detected accurate regions of changes with aging using the DARTEL algorithm for the first time. Atrophic regions with aging revealed in the present study will be important information to discuss whether the detected volume changes in a clinical or experimental setting occurred by acquired disease or natural aging effects.

The regional aging analysis in the present study revealed a decrease in the bilateral parietal cortex. The decreased parietal cortex in cats may correspond with Brodmann areas 1–3, 5, and 7 in humans (31). Brodmann areas 1–3 in humans are the primary somatosensory cortex responsible for perceiving touch, pain, temperature, and proprioception (32), and Brodmann areas 5 and 7 are the parietal association cortex, which has various functions related to visual, auditory, and somatosensory information (33). In cats, these areas form a connective network with each other (34). In a feline study investigating the brain regions corresponding to the pain and the efficacy of remifentanil using functional MRI, the somatosensory area, parietal association area, cingulate cortex, hippocampus, and cerebellum were activated by mechanical noxious stimulation (35). Since the somatosensory area receives an impulse from the nociceptor, atrophy in the parietal cortex caused by neural loss and/or degeneration may relate to pain obtundation in aging cats. In addition, the feline cingulate cortex creates networks with the parietal association cortex and the pyriform cortex (34, 36). The cingulate cortex is thought to implicate sleep quality or cycle because it has been reported that volume loss of the right posterior cingulate cortex was associated with poor sleep quality in healthy humans (37). Since the primary somatosensory cortex and the parietal association are connected intricately with the surrounding brain area including the cingulate cortex, the decreased regions in the present study might have been detected as one large cluster. Generally, it is considered that the limbic system including the cingulate cortex, the amygdala, and the hippocampus is implicated in various functions such as emotion, learning, and memory. Although bilateral resection of the pyriform lobes containing the amygdaloid complex in cats has caused behavior and sexual changes similar to Klüver-Bucy syndrome in humans (38), cats have not shown these abnormalities when the resection is unilateral (39). For this reason, the onset of clinical signs of the feline limbic system may be determined by the severity of functional deficits, as well as the amount of neuronal loss. In VBM analysis, investigating the relationships between the onset of neurological and/or behavioral abnormality and the severity of morphometric changes is required.

In the present study, decreased global volume with aging in cats was observed in the GM and the total brain. According to these results, the total brain volume decrease may be caused by decreased GM volume, particularly in the parietal cortex, since there was no association between volume and aging in the WM. In the aged canine brain, magnetic resonance findings have been described such as cortical atrophy, ventricular dilation, and fissural and sulcal enlargement (24, 40, 41). However, the region of interest method requires serial examinations, expert subjective evaluations, and/or several time-consuming measurements (42). On the other hand, a canine aging study using VBM analysis has reported that age-related GM reductions were observed in the parietal and temporal lobes, thalamus, cerebellum, and brainstem (11). Although the VBM method reveals a more detailed atrophied area than the region of interest method, an individual analysis may decrease the detection accuracy in the VBM method. For these reasons, measurement of the interthalamic adhesion size on MRI has been introduced clinically as criteria for brain atrophy with or without cognitive dysfunction in dogs (42–44). To the best of our knowledge, no report has compared the interthalamic adhesion size in cats with cognitive dysfunction with that of healthy cats. The interthalamic adhesion is the prominent structure that connects the left and right thalamus and is easy to quantitate in cats, similar to dogs. The present study using cats without any neurological and behavioral signs did not show a significant age-related reduction in the interthalamic adhesion. There is a possibility that it is useful to evaluate interthalamic adhesion when cats with cognitive dysfunction show small interthalamic adhesion size.

Pathological reviews for feline brain aging have shown age-related alterations such as neuronal loss and atrophy, amyloid-β deposition, and tau phosphorylation (1, 16). It may be capable of detecting subtle structural changes caused by these pathological alterations since the VBM analysis is an objective method to detect decreased regions using magnetic resonance signal intensity and volume for each subject. However, our results did not correspond completely with anatomical regions observed in these pathological alterations. In the brains of old cats, amyloid-β deposition and tau phosphorylation have been observed in not only the parietal cortex, but also the parahippocampal cortex and occipital cortex (45). Additionally, neuronal loss in old cats has been observed in the hippocampus (14), caudate nucleus (46), and cerebellum (47). In particular, Chambers et al. (14) reported that domestic cats >14 years old naturally accumulate amyloid-β in the neuropil throughout the cerebral cortex, produce neurofibrillary tangles in the hippocampus, and suffer hippocampal neuronal loss. For detecting a small localized abnormality in the feline using VBM analysis, it may be necessary to modify specific parameters including spatial resolution, segmentation, normalization, and smoothing. The number of evaluated voxels in each region is low in the VBM analysis since the brain size of cats is smaller than that of humans and dogs. Regions that do not show a significant decrease in the present study might also have pathological changes that are undetectable by VBM analysis.

A significant limitation of the present study is that the study design is retrospective. From medical records, the statistical analysis in the present study included 65 cats, with 15 of the cats > 12 years old. Cats are considered old if >12 years old (16), and cats > 15 years old are considered super senior (1). Age-related problems in cats often begin with changes in behavior, such as excessive vocalization, spatial and/or temporal disorientation, alterations in interactions with owners or other pets, alterations in the sleep-wake cycle, house soiling, alterations in activity levels, anxiety, and/or learning and memory deficits (1). However, these behavioral abnormalities without other medical problems also may not be reported by the owners of aged cats (48). Although the inclusion criteria of the present study were that cats have no apparent neurological and behavioral signs, the cats were not subjected to any neurobehavioral evaluation and were not assessed by veterinary behaviorists. Since owners of senior cats may not recognized age-related behavioral changes as abnormal signs, neurological and behavioral changes may originate from the functional deficits of the parietal lobe. Although we cannot conclude whether the atrophied parietal cortex in the present study causes feline cognitive dysfunction syndrome, aging cats with cognitive dysfunction may show significant atrophy in those regions. Further study, including cases with behavioral changes or cognitive dysfunction, is required to clarify these issues.

As mentioned above, VBM analysis is used rarely in veterinary medicine. For accurate VBM analysis, each subject must have low variation in brain shape. In particular, there is remarkable difference in the brain parenchyma and ventricular shape between dog breeds. Therefore, several brain templates were developed according to head shapes (49). In contrast, many cat breeds have similar brain shapes, except for brachycephalic breeds such as Exotic shorthair, Persian, and Himalayan. Accordingly, feline VBM analysis may be a reproducible and accurate measurement compared with that of canine. Recently, the life span of pet cats has been increasing because of advances in veterinary medicine, improvements in nutrition, and environment management (1). Increasingly, the clinician may provide appropriate veterinary care for cats with age-related behavior problems, but the diagnosis of cognitive dysfunction syndrome is challenging since it is made only by ruling out other potential causes. When cognitive decline or dysfunction is caused by age-related morphological changes, visually detectable changes, as observed in the present study, may be important. Therefore, we consider that VBM analysis should be introduced widely in the feline veterinary field.

## Data availability statement

The raw data supporting the conclusions of this article will be made available by the authors, without undue reservation.

## Ethics statement

The animal study was reviewed and approved by the Animal Care and Use Committee of Nippon Veterinary and Life Science University. Accession numbers: 25K-4, 25K-6, 26K-27, 26K-29, 27K-8, 27K-10, 28K-2, 28K-4, 29K-4, 29K-5, 30K-4, 2019K-2, 2020K-3, 2021K-2; representative researcher is DH. Written informed consent was obtained from the owners for the participation of their animals in this study.

## Author contributions

YH and DH conceived and designed the present study. YH, YY, RA, and SM collected the data. YH analyzed the data and wrote the draft manuscript. YY, RA, SM, and DH performed the critical revisions of the manuscript. All authors approved the final manuscript.

## Funding

This work was supported partially by the JSPS KAKENHI (Grant Number 19K16005; Principal investigator is YH).

## Conflict of interest

The authors declare that the research was conducted in the absence of any commercial or financial relationships that could be construed as a potential conflict of interest.

## Publisher's note

All claims expressed in this article are solely those of the authors and do not necessarily represent those of their affiliated organizations, or those of the publisher, the editors and the reviewers. Any product that may be evaluated in this article, or claim that may be made by its manufacturer, is not guaranteed or endorsed by the publisher.

## Abbreviations

CSF: cerebrospinal fluid
DARTEL: diffeomorphic anatomical registration using exponentiated lie algebra
GM: gray matter
MRI: magnetic resonance imaging
SPM: statistical parametric mapping
VBM: voxel-based morphometry
WM: white matter.
